# Impact of Liposomal Drug Formulations on the RBCs Shape, Transmembrane Potential, and Mechanical Properties

**DOI:** 10.3390/ijms22041710

**Published:** 2021-02-08

**Authors:** Sylwia Cyboran-Mikołajczyk, Przemysław Sareło, Robert Pasławski, Urszula Pasławska, Magdalena Przybyło, Kacper Nowak, Michał Płóciennik, Halina Podbielska, Marta Kopaczyńska, Magdalena Wawrzyńska

**Affiliations:** 1 Department of Physics and Biophysics, Faculty of Life Sciences and Technology, Wrocław University of Environmental and Life Sciences, Norwida 25, 50-375 Wrocław, Poland; sylwia.cyboran@upwr.edu.pl; 2 Department of Biomedical Engineering, Faculty of Fundamental Problems of Technology, Wrocław University of Science and Technology, Wybrzeże Wyspiańskiego 27, 50-370 Wrocław, Poland; przemyslaw.sarelo@pwr.edu.pl (P.S.); magdalena.przybylo@pwr.edu.pl (M.P.); halina.podbielska@pwr.edu.pl (H.P.); marta.kopaczynska@pwr.edu.pl (M.K.); 3 Department of Veterinary Surgery, Institute of Veterinary Medicine, Faculty of Biological and Veterinary Sciences, Nicolaus Copernicus University in Toruń, Gagarina 11, 87-100 Toruń, Poland; r.paslawski@umk.pl; 4 Department of Diagnostics and Clinical Sciences, Institute of Veterinary Medicine, Faculty of Biological and Veterinary Sciences, Nicolaus Copernicus University in Toruń, Gagarina 11, 87-100 Toruń, Poland; urszula.paslawska@umk.pl; 5 Department of Internal Diseases and Clinic of Diseases of Horses, Dogs and Cats, Faculty of Veterinary Medicine, Wroclaw University of Environmental and Life Sciences, Norwida 25, 50-375 Wrocław, Poland; kacper.nowak@upwr.edu.pl; 6 Department of Biochemistry and Molecular Biology, Faculty of Veterinary Medicine, Wroclaw University of Environmental and Life Sciences, Norwida 25, 50-375 Wrocław, Poland; michal.plociennik@upwr.edu.pl; 7 Department of Preclinical Studies, Faculty of Health Sciences, Wrocław Medical University, Wybrzeże Paustera 1, 50-367 Wrocław, Poland

**Keywords:** shape of erythrocytes, anisocytosis, Photolon, phosphatidylcholine liposomes, monolayer liposome formulations, biomechanical properties, transmembrane potential, osmotic resistance, drug carrier

## Abstract

Liposomal technologies are used in order to improve the effectiveness of current therapies or to reduce their negative side effects. However, the liposome–erythrocyte interaction during the intravenous administration of liposomal drug formulations may result in changes within the red blood cells (RBCs). In this study, it was shown that phosphatidylcholine-composed liposomal formulations of Photolon, used as a drug model, significantly influences the transmembrane potential, stiffness, as well as the shape of RBCs. These changes caused decreasing the number of stomatocytes and irregular shapes proportion within the cells exposed to liposomes. Thus, the reduction of anisocytosis was observed. Therefore, some nanodrugs in phosphatidylcholine liposomal formulation may have a beneficial effect on the survival time of erythrocytes.

## 1. Introduction

Liposomal formulations have been proven to possess several advantages among others, as potential drugs carriers [1]. Plenty of research experiments in the field of liposomes have been carried out and their applications are well established in various areas. To the market of medicinal pharmaceutics various combinations have been introduced, among others, liposomal formulations of cytotoxic drugs, antibiotics, analgesics or drugs used in photodynamic therapy [1,2,3,4]. Moreover, liposome technologies are regarded as one of the key technologies for improving existing therapies with insufficient efficacy and/or a high degree of side effects. This could become possible by liposome enrichment of particular molecules to immobilize the desired drug in the carrier, more efficiently [5] or to enable liposomal formulation of drug to cross the body’s natural barriers, which is impossible for the drug itself [6], as well as simultaneous packaging and delivery to the target site of two or more drugs to enhance the therapeutic effect of co-therapy [7]. These facts influenced the emergence of a new group of drugs, so-called super generic drugs, i.e., drugs that differ from the original drugs in their pharmaceutical formulation. In this way, new, more favorable therapeutic properties can be achieved, while the active ingredient remains the same [3]. Nowadays, the adjuvant properties of liposomal formulations have been used and were exploited for liposome-formulated RNA vaccines development for preventing infectious diseases or treating some cancers, which in the clinical trials have been showed to be safe and well-tolerated [8,9,10].

Liposomes are one of the types of nanodrug delivery systems, where the active substance is delivered with the use of self-assembling materials that form the vesicle nanostructures [1]. Targeted Drug Carriers (TDC) raise particular hopes for low-molecular-weight drugs, the properties of which necessitate a change in pharmacokinetics. These drugs are usually toxic substances, which after systemic administration (especially orally or most often, intravenously), reach as well affected area as healthy cells. This results in a large volume of drug distribution and a low therapeutic index. Placing the drug in an appropriately selected macromolecular carrier like liposomes, leads to a very significant increase in the concentration in the target affected area, but not in healthy tissues. The liposomal drug remains in plasma for longer time and the volume of distribution is smaller [2]. These beneficial effects are a result of a phenomenon referred to as Enhanced Permeability and Retention (EPR). EPR is based on the fact that nanodrugs, such as liposomal forms of active substances tend to selectively accumulate in the tissues of solid tumors due to the special properties of blood vessels supplying neoplasms with blood [11,12,13].

Liposomes, whose lipid bilayer consists mainly of phospholipids, with a surface not modified (for example with polymers) are very effectively removed by the mononuclear phagocyte system (MPS). Therefore, they constitute a potentially very good carrier for macrophage-targeting substances. Literature data show that both, liposomes smaller than 100 nm and larger, are effectively captured by these cells [14]. It has been proven that multilamellar liposomes, which were formed by means of 200–450 nm filters, when delivered intravenously, significantly decreased the liver macrophages phagocytic index (by approximately 50%), comparing to small unilamellar liposomes [15,16,17]. The endocytosis efficiency depends on additional parameters of the liposomes, such as the size, surface charge and micromechanics of the lipid bilayer. Due to the high observed systemic toxicity of positively charged liposomes, solutions where the surface charge is neutral or negative, are definitely preferred. The latter is characterized by a particularly high degree of macrophage recognition [14].

Targeted therapies using nanocarriers to deliver the drugs into lesion, are currently widely studied. Very often such drugs immobilized in nanocarriers are administrated intravenously [18]. Liposomes have been shown to have different influence on red blood cells (RBCs) [19,20,21]. They minimize membrane damage occurring during 42-d hypothermic storage [22,23]. The protective effect of liposomes has been attributed to membrane modifications. However, some liposomes (e.g., pegylated arsonoliposomes) caused significant hemolytic effect [24]. Therefore, the search for drugs composed of nanolipids must begin from assessing their effect on membranes of RBCs. The aim of the present study was to determine the effect of large unilamellar vesicles (LUVs) containing Photolon (a photosensitizer composed of the sodium salt form of chlorin e6) on selected physical properties of erythrocytes in pigs fed a standard diet. In our study Photolon was used as an example of hydrophobic, water-insoluble molecules, that require a carrier. Additionally, its fluorescence served better visualization. The liposome formulations composed of phosphatidylcholine (PC)—P1, liposome formulations composed of PC and cholesterol (30%)—P2—and liposome formulations composed of PC, cholesterol (30%), and polymer polyethylene glycol (PEG)—P3, were assessed as the most common, currently studied formulations, where cholesterol is responsible for sealing the LUVs, while PEG increases the time the LUVs circulate in the body [25,26].

Moreover, according to our previous study, where the excellent physical and pharmacokinetic properties and selectivity for macrophages were proven and these formulations were regarded as a promising therapeutic candidate for use in arteriosclerosis treatment [4], we decided to examine whether photodynamic active compound encapsulated in liposome formulation may evoke changes in RBCs morphology, biomechanics, and physiology.

## 2. Results

### 2.1. Liposome Characterization: Size Distribution and Encapsulation Efficiency

**Figure 1:**
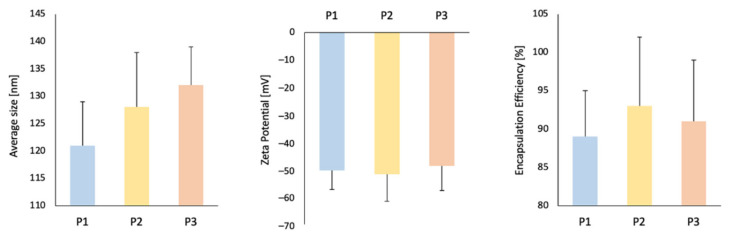
Average size ± SD, Zeta Potential ± SD and encapsulation efficiency ± SD (EE) for appropriate liposomal formulation.

All the analyzed liposome formulations are characterized by an average size in the range of 113–139 nm, which is consistent with the measurements carried out by means of transmission electron microscopy (to further compare). Polydispersity indexes are without significant changes within proposed formulations, as well as zeta potential. Moreover, the Photolon encapsulation efficiency is around 90%, what can be regarded as an effective method of closure the desired molecules (in this case, a Photolon) inside the liposomal carrier (LUVs)—Table 1 and Figure 1. There were no statistically significant differences in the encapsulation efficiency of Photolon in the assumed formulations, therefore, the unmodified by cholesterol or PEG formulation (i.e., P1 formulation) was considered to be the most promising for its properties.

### 2.2. Electron Microscopy Visualization of Liposomal Formulation

**Figure 2:**
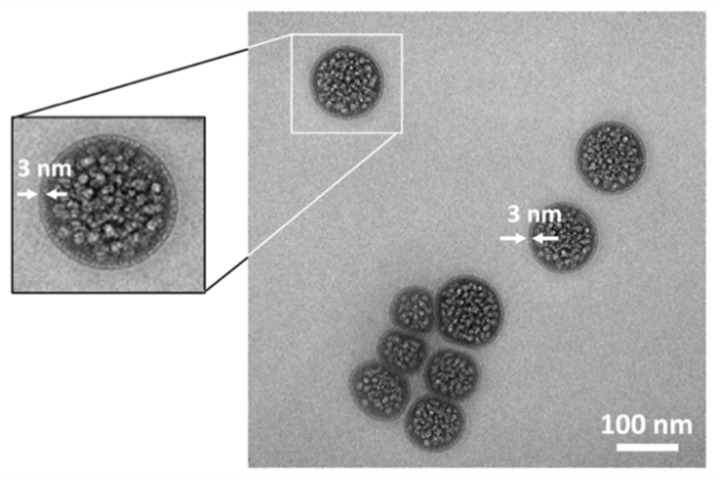
Transmission electron micrographs of P1 liposomal formulation of Photolon, where photosensitizer is uniformly distributed inside of nanovesicle. The single spheroidal lipid bilayer is about 3 nm thick.

The liposomal formulations were visualized by transmission electron microscopy. As a representative, the TEM images of P1 formulation are presented in Figure 2. The images depict the presence of large number of nanovesicles, which are loaded with Photolon. The distribution of the Photolon molecules inside of liposomes is uniform. The average size of liposomes is approximately 100–150 nm, where the external layer of liposome consisted of single spherical lipid bilayer is about 3 nm thick, as it is expected for single lipid bilayer [27]. The results are consistent with those obtained by means of DLS.

### 2.3. Photolon Liposomal Formulations Hemolytic Activity

**Figure 3:**
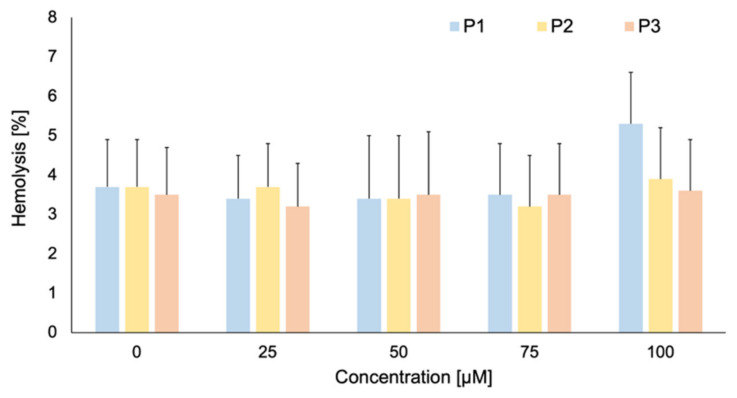
The percentage of hemolyzed red blood cells (RBCs) under the influence of P1, P2, and P3 liposomal formulation at 10–100 µM concentration.

The hemolytic activity examinations have shown that each of the studied Photolon liposomal formulation do not induce hemolysis of RBCs—the hemolysis does not exceed 5% (Figure 3). There are no significant differences between no treated RBCs in comparison to RBCs treated by the particular liposomal formulation. For the further studies, the 50 µM concentration of liposomal formulation, was chosen.

### 2.4. RBCs Osmotic Resistance Changes under Liposomal Formulation Impact

**Figure 4:**
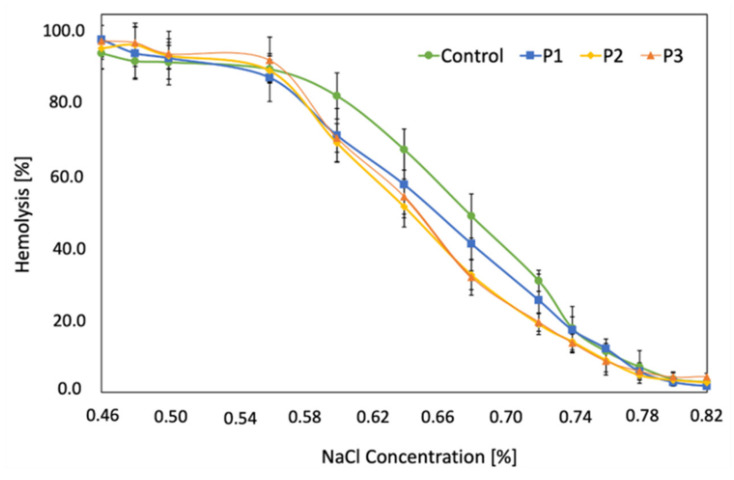
Hemolytic curves of control and cells interacted with P1, P2, and P3 liposome formulations used at a concentration of 50 μM.

The tested Photolon liposomal formulations have varying impact on the RBCs osmotic resistance in vitro. The relationships between the percentage of hemolysis of control and LUVs modified blood cells and NaCl concentration, i.e., hemolytic curves are presented in Figure 4. Modification of the RBCs membrane with the P1 formulation did not significantly change its osmotic resistance as evidenced by a slight shift in the hemolytic curve towards lower NaCl concentrations. Nevertheless, RBCs incubated with P2 and P3 formulations showed slightly (statistically significant at α = 0.1) higher changes in osmotic resistance. The NaCl (C_50_) concentration at which 50% of RBCs were hemolyzed determined from plotted relationships is: 0.68 ± 0.15%, 0.66 ± 0.14%, 0.64 * ± 0.11% and 0.65 * ± 0.10%, for control cells and cells affected by P1, P2, and P3, respectively (statistically significant differences between control and P1, P2, or P3 were marked with *).

### 2.5. RBCs Transmembrane Potential Changes upon LUVs Modification

The fluorometric studies exploiting the DiSC3(5) fluorescence indicator, allowed for the determination of the membrane potential of the control RBCs and RBCs influenced by formulations P1, P2 and P3, in concentrations of 50 µM. The determined values of transmembrane potential are presented in Table 2. The obtained results indicate that RBCs treated with P1 and P2 liposomal formulations have a more negative value of membrane potential (statistically significant at α = 0.05), whereas the potential of cells treated with P3 formulation does not differ significantly from the membrane potential of the control erythrocytes (difference is not statistically significant at α = 0.05).

### 2.6. Biomechanical Parameters of RBCs upon LUVs Interaction

Optical tweezers were used to measure the RBCs stiffness. It has been shown that the erythrocytes treated with the formulations change their mechanical properties and are more flexible (less rigid) as it is depicted by Table 3. This is particularly evident in relation to the erythrocytes treated with the P1 and P2 formulations. These erythrocytes are more flexible comparing to control (statistical significance at α = 0.05). In the case of P3 interacted RBCs, a change in the biomechanical parameters is also observed, but to a lesser extent (difference is not statistically significant at α = 0.05).

### 2.7. Microscopic Evaluation of the RBCs Shape

**Figure 5:**
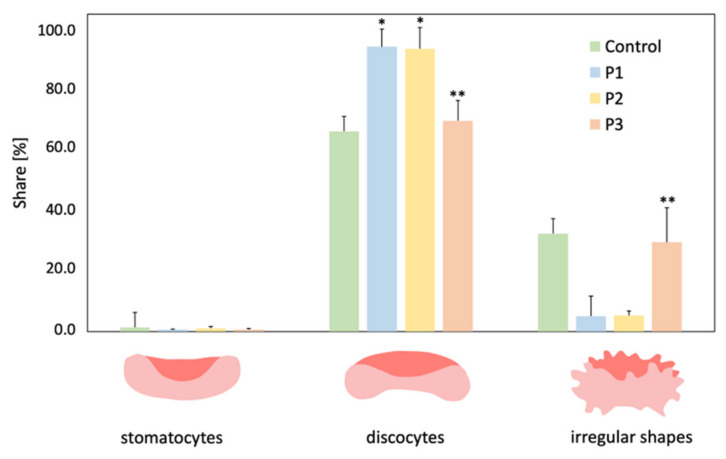
Percentage share of shape of RBCs modified by interaction with formulations P1, P2, and P3 in concentration 50 µM and control. * Statistically significant differences between control and P1, P2 or P3-treated cells are denoted (α = 0.05). ** Statistically significant differences between P3 and P1 or P2 treated cells (α = 0.05).

The interaction of RBCs with the tested Photolon liposome formulations revealed that examined formulations caused various degree changes in the RBCs shape. The interaction of P1 and P2 formulations increased the number of discocites and decreased the number of irregularly shaped cells (echinocytes, pancake-shaped RBCs, and others) comparing to the control cells (statistically significant at α = 0.05). As can be seen in the Figure 5, the erythrocytes after treatment with P1 and P2 changed the shape comparing to the control and mostly discocites were observed and these ones with irregular shapes have lower share. In addition, no statistically significant differences at α = 0.05 were found between the effect of P1 and P2 formulations on RBCs shapes. The shapes of RBCs interacted with P3 formulation did not differ significantly from untreated RBCs, but significantly differed from P1 and P2 (statistically significant at α = 0.05).

### 2.8. Confocal Microscopy Study of RBCs-LUVs Photolon Redistribution

**Figure 6:**
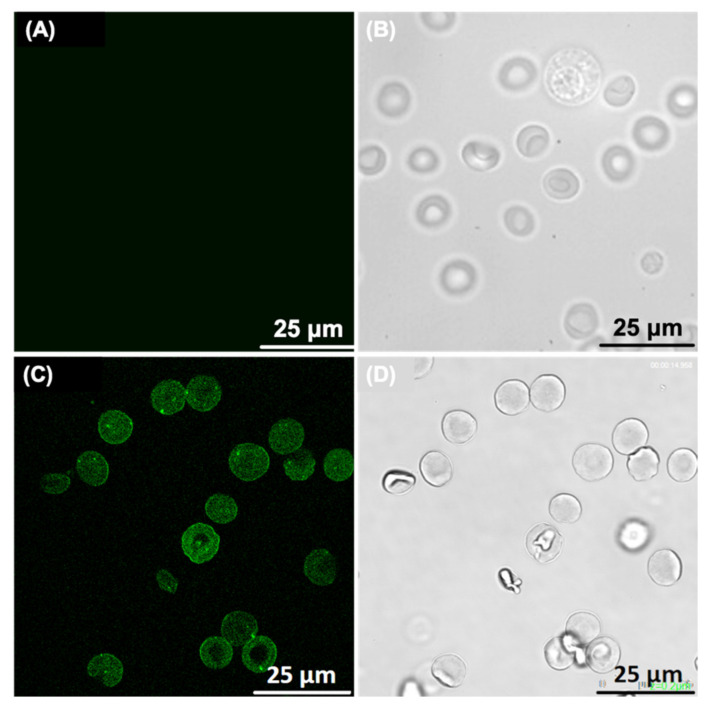
(**A**,**B**) confocal microscopy images of control RBCs—without liposomal formulation (fluorescence—(**A**) and transmission (**B**)—mode). (**C**,**D**) confocal microscopy images of the RBCs influenced by P1 liposomal formulation of Photolon (fluorescence—(**C**) and transmission (**D**)—mode).

The confocal microscopy study was performed in order check whether the Photolon is accumulated within the RBCs (Figure 6).

The above observations correlate with liposomal distribution within RBCs visualized with the use of confocal microscopy. The P1 liposomal formulation penetrates into erythrocytes, mainly in the peripheral parts of the cells and no Photolon is observed in the central part of the RBCs (what corresponds to the concave, characteristic feature of the normal erythrocytes). The lower accumulation of the photosensitizer within central part of the RBC it may simply be associated with a reduced amount of cytoplasm and the occupation of this area of RBC by hemoglobin, which naturally fills a significant fraction of this blood cell.

### 2.9. Statistical Analysis

Statistical analysis of the obtained results was performed using the STATISTICA 12.0 (StatSoft Polska, Kraków, Polska) software. Depending on the experiment, the following tests were used: the Kolmogorov–Smirnov test (normality test), the *t*-test (for independent samples), and Dunnett’s post-hoc ANOVA test at significance level α = 0.01 or α = 0.05. The *t*-test was used to estimate the differences between mean values of measured parameters. Dunnett’s test was used to estimate the differences between mean value of measured parameters of control samples and mean value of the parameters for samples affected by the Photolon formulations. All the experiments were done in at least five replicates, the results being presented as means ± standard deviation (SD).

## 3. Materials and Methods

### 3.1. Liposome Preparation Protocol

The purity of phosphatidylcholine from soya beans (Phospholipon^®^ 90G) (Lipoid AG, Steinhausen, Switzerland) was checked by high-performance liquid chromatography (HPLC, Knauer, Berlin, Germany) equipped with an evaporative light scattering detector (ELSD, Alltech 3300, Buchi, Flawli, Switzerland) as recommended by the manufacturer and validated in our laboratory, according to The Organisation for Economic Co-operation and Development (OECD) and International Council on Harmonisation of Technical Requirements for Registration of Pharmauceuticals for Human Use (ICH) requirements. Phosphate buffered saline (PBS) were prepared from PBS tablets (Sigma-Aldrich, Saint Louis, MO, USA) and were filtered through 200 μm filter (Whatman, Maidstone, UK) after preparation. Conductivity and pH of solutions were checked before each experiment. Photolon (Belmedpreparaty, Minsk, Belarus) was used as a pharmaceutical ingredient. Liposomes prepared by the gel hydration method (EUP 17 162 568.4) at high concentrations (>20% *w*/*w*), were dissolved in propylene glycol (Sigma-Aldrich, Saint Louis, MO, USA) and hydrated with aqueous phase containing Photolon. Next, the final liposomal gel was extruded 5 times through a 100 nm pore size polycarbonate Whatman^®^ filter. The final liposome suspension consists of 20% *w*/*w* Phospholipon^®^ 90G, 25% *w*/*w* of propylene glycol, 49.55% *w*/*w* water and 0.45% *w*/*w* of Photolon. Subgroup P1 liposome formulations were composed of phosphatidylcholine, P2 was composed of phosphatidylcholine and cholesterol, P3 was composed of phosphatidylcholine, cholesterol, and polymer polyethylene glycol.

### 3.2. Size Distribution and Zeta Potential Measurements of the Photoactive Liposomal Formulation

The liposome’s size and zeta-potential were determined using the dynamic light scattering (DLS) technique (Malvern Zetasizer Nano ZS, Malvern Instruments, Malvern, UK). The instrument was equipped with a He-Ne laser emitting at 633 nm, a measurement cell, a photomultiplier and a correlator. The samples were diluted around 100 times with the buffer, so the sample osmotic balance was maintained, and placed in 1 cm polystyrene cells. The refractive index of the material and viscosity were fixed to 1.33 and 0.9025 cP, respectively. The scattering intensity was measured at 25 °C at a scattering angle of 173° relative to the laser source. The liposome size was derived from the correlation function using software provided with the instrument. Each measurement was performed three times with 12 repetitions.

### 3.3. Encapsulation Efficiency of the Photoactive Liposomal Formulation

The encapsulation efficiency was determined using ultrafiltration method. Samples of liposomal gels were diluted at least 5 times before ultrafiltration by means of PS MicroKros Filter Modules (C02-E050-05-N, Spectrum Labs, San Francisco, CA, USA) composed of 20 cm polysulfone tubes with 30 kDa cutting size pores. Three successive ultrafiltrations were performed on each sample to make sure the extravascular polymer was removed. A small fraction of permeate (about 1 mL), as well as initial solutions and retentate, were collected for subsequent evaluation. The evaluation of Photolon quantity inside liposomes was determined basing on fluorescence measurements following the destabilization of lipid bilayer by the addition of 100 µL of Triton X-100 (Sigma-Aldrich, Saint Louis, MO, USA) at 20%. The encapsulation efficiency was calculated according to the following formula: EE = C^in^/C^tot^, where C^in^ and C^tot^ are Photolon concentration inside liposomes and total Photolon concentration, respectively.

### 3.4. Electron Microscopy Visualization of Liposomal Formulation

The transmission electron microscopy (TEM) images of liposomes with Photolon were obtained using Tecnai G2 20 X-TWIN (FEI Company, Hilisboro, Oregon, USA) transmission electron microscope equipped with field emission gun, operating at an acceleration voltage of 200 kV. Images were recorded by means of 4k × 4k Eagle CCD camera with high sensitivity (HS) scintillator (FEI Company, Hilisboro, OR, USA) and processed with TIA software (FEI Company, Hilisboro, OR, USA). The samples were placed onto a grid covered with a collodion film. Then, measurements were performed with staining solutions containing 1% of phosphotungstate, which was blotted off after 60 s following sample deposition.

### 3.5. Animals and Blood Samples Collection

The experiment was performed with the approval No. 37/2015 of the first Local Ethics Committee for Animal Experimentation of the Institute of Immunology and Experimental Therapy in Wrocław, Poland. The blood samples were taken from the nine healthy Polish White female pigs (*Sus scrofa*) (approximately 40 kg body weight). Pigs were housed in a single room, divided into individual pens, with a temperature of 18–20 °C and 60–75% humidity. Swine were fed with standard diet and had unlimited access to water. Before each blood sample donation pigs fasted 12 h but had free access to the water. The blood samples were collected into a K3EDTA tube (KABE Laboratechnik, Nümbrecht, Germany) by ear vein puncture.

### 3.6. Photolon Liposomal Formulations Hemolytic Activity Examination

The hemolytic activity of P1, P2 and P3 samples was assessed. For washing the RBCs, and in the performed experiments, an isotonic PBS of pH 7.4 (131 mM NaCl, 1.79 mM KCl, 0.86 mM MgCl_2_, 11.79 mM Na_2_HPO_4_∙2H_2_O, 1.80 mM Na_2_H_2_PO_4_∙H_2_O—all from Avantor Performance Materials, Gliwice, Poland), was used. Upon their removal from plasma, the RBCs were washed four times in PBS. Then, the LUVs incubation of RBCs was conducted at 37 °C for 1 h, each sample containing 1 mL of RBCs suspension of 1.2% hematocrit (HCT), as well as appropriate amounts of the formulations studied (the final concentrations were in the range 10–100 µM) and stirred continuously. After this process to each sample 2 mL of PBS, was added. Next, samples were centrifuged, and the supernatant assayed for hemoglobin content using a spectrophotometer (Spekord 40, Analityk Jena AG, Jena, Germany) at 540 nm wavelength. HGB concentration in the supernatant, expressed as percentage of HGB concentration in the supernatant of totally hemolyzed cells, was assumed as the measure of the extent of hemolysis.

### 3.7. Assessment of the Osmotic Resistance of RBCs Exposed to Liposomal Formulations

Osmotic resistance assay was performed. Full blood was centrifuged for 3 min, 2500 rpm at 4 °C, to remove the plasma and white blood cells (WBCs). The RBCs obtained were washed thrice with a cool (ca. 4 °C), 310 mOsm PBS isotonic solution. Next, 1.2% RBCs suspension containing 50 µM of LUVs was prepared and left for 1 h at 37 °C with continuous stirring. After this incubation, the suspension of RBCs was centrifuged for 15 min at room temperature in order to remove the cells from the compound solution. 100 µL RBCs sediment was taken and suspended in test tubes containing NaCl solutions in 0.5–0.86% concentration range and an isotonic (0.9%) NaCl solution. Then, the suspension was stirred and centrifuged. After that, the percentage of hemolysis was measured with a spectrophotometer at 540 nm wavelength. Based on the results obtained, the relation was determined between the percentage of hemolysis and NaCl concentration in the solution. Next, using obtained plots, the NaCl percent concentrations (C_50_) that caused 50% hemolysis was found. The C_50_ values were taken as a measure of osmotic resistance. If a determined sodium chloride concentration is higher than that of control cells, the osmotic resistance of the RBCs is regarded to be lower, and vice versa.

### 3.8. Erythrocytes Transmembrane Potential Measurement

The transmembrane potential of RBCs was measured with the fluorescence indicator 3,3′-Dipropylthiadicarbocyanine iodide [DiSC3(5)] (Sigma-Aldrich, Saint Louis, MO, USA) [28]. For in vitro experiment, 1 mL of RBCs solution of 2% HCT was incubated with 50 µM of formulations for 1 h at 37 °C. Then, 2 mL of 310 mOsm PBS was added to the suspension, centrifuged for 15 min at room temperature and the supernatant removed, in order to separate the cells from the LUVs solution. Next, 220 µL of thus prepared RBCs were suspended in PBS, containing 10 mM Tris-HCl, pH 7.4 and 150 mM (KCl and NaCl), with K^+^ concentration increasing from 50 to 140 mM. Next, an ethanolic solution of DiSC3(5) was added to final concentration of 2 mM, and samples were incubated for 10 min at room temperature. The fluorescence intensity of the dye was measured with a Varian Cary Eclipse fluorescence spectrophotometer (Agilent Technologies, Santa Clara, CA, USA) at 660 nm and excited at 625 nm. Then, the ionophore valinomycin (Sigma-Aldrich, Saint Louis, MO, USA) was added to the samples to final concentration of 1 mM, incubated for 10 min and fluorescence intensity of the probe was measured again. The external potassium concentration for which no change in DiSC3(5) fluorescence intensity occurs upon valinomycin addition, was calculated. The transmembrane potential was calculated from Nernst equation for monovalent ions, where intracellular potassium concentration of erythrocytes was 152 mM.

### 3.9. Biomechanical Parameters of RBCs

In order to study the mechanical properties of erythrocytes, holographic optical tweezers were used equipped with PLUTO-NIR refractive modulator (HOLOEYE Photonics, Berlin, Germany) for creating different optical traps at the same time. The laser source was Ventus (Laser Quantum, Stockport, UK), which delivers 4 W of optical power at wavelength of 1064 nm, where power was limited to a needed value by a variable attenuator. The optical traps were created by focusing laser beam by a microscopic lens with high numerical aperture. The system was built on an IX71 inverted microscope (Olympus, Tokyo, Japan). For the experiment, 1 mL of RBCs solution of 1.2% HCT was incubated with 50 µM of formulations for 1 h at 37 °C. After that, erythrocytes were suspended in PBS. In order to perform the cell surface biotinylation, 1.5 mg EZ-LinkLM Sulfo-NHS-LC-LC-Biotin (Thermo Fisher Scientific, Waltham, MA, USA) was added per 1 mL of the PBS suspended erythrocytes. After 30 min of incubation in 37 °C cells were thrice washed in PBS and afresh suspended in PBS. Subsequently, to the sample streptavidin-coated polystyrene microbeads (Spherotech, Lake Forest, IL, USA), were added. The experiment comprised measuring the force necessary to stretch the erythrocytes by 1 µm.

### 3.10. Shape of RBCs Assessment

For this investigation, RBCs obtained from healthy pigs were separated from plasma and washed four times in PBS (Sigma-Aldrich, Saint Louis, MO, USA). Next, they were suspended in the same solution, but containing 50 µM of the formulations studied. RBCs in the 1.2% solution per 1 h at 37 °C, were fixed with a 0.2% solution of glutaraldehyde (Avantor Performance Materials, Gliwice, Poland). After that RBCs were observed under optical microscope (Nikon Eclipse E200, Minato, Tokyo, Japan) equipped with a digital camera. The obtained images made it possible to count RBCs of various shapes, and then the percent share of the two basic forms (echinocytes and stomatocytes) in a population of ca. 800 cells, was determined. Individual forms of RBCs were divided into three groups: stomatocytes, discocites and cells with irregular shape (echinocytes, pancake-shaped RBCs, and other irregular shape).

### 3.11. Confocal Microscopy of Erythrocytes

For examination of the erythrocyte’s shape and morphology by means of confocal microscopy, as well as the Photolon redistribution between RBCs and drug carriers (i.e., LUVs), RBCs were separated from plasma and washed three times in PBS. RBCs were suspended in the PBS containing 50 µM of the liposomal formulations studied. Then, RBCs were incubated for 1 h at 37 °C. Afterwards, the cells were placed on a microscope slide, covered by microscope cover slip and examined by means of confocal microscope (Leica TCS SPE, Leica Microsystems, Wetzlar, Germany) supported by LAS AF software (Leica Microsystems, Wetzlar, Germany). The fluorescence images were obtained with wavelength of 405 nm, which is close to the excitation maximum of Photolon. The detection of the Photolon emission signal occurred at approximately 670 nm wavelength.

## 4. Conclusions

In this work, the impact of liposome formulations composed of phosphatidylcholine (P1), phosphatidylcholine and cholesterol (P2), and phosphatidylcholine, cholesterol, and polymer polyethylene glycol (PEG) (P3) on shape, transmembrane potential, biomechanical parameters and osmotic resistance of erythrocytes, were assessed. The phosphatidylcholine formulations as well as cholesterol-modified formulations (in order to seal liposomes) or PEG-functionalized liposomes (to ensure longer circulation time in the body) were evaluated. It was shown that erythrocytes treated with P1 and P2 formulation are characterized by a lower value of transmembrane potential and stiffness, which also correlates with higher hemolytic resistance and a reduced number of stomatocytes and cells with irregular shapes. From the other hand, a visible increase of discocites number, was observed. As a result, a decrease in erythrocyte anisocytosis was noted through the interaction of erythrocytes with phosphatidylcholine formulations. The presence of PEG in the P3 liposome formulation limits the adhesion of liposomes to erythrocytes, which results in the lack of or slight changes in relation to changes in the shape, stiffness, or transmembrane potential of erythrocytes. Moreover, the P1 formulation has shown to improve RBCs shape conditions to a better extend than other formulas composed of additional cholesterol and PEG fillers. Therefore, nanodrugs in liposomal formulations of the P1 type may have a beneficial effect on the survival time of erythrocytes. Such liposome-based carriers forms should be considered for clinical use in drug delivery systems.

## Figures and Tables

**Table 1:** Average size ± SD, polydispersity index (PDI), zeta potential ± SD and encapsulation efficiency ± SD (EE).

	Average Size [nm]	PDI [–]	Zeta Potential [mV]	EE [%]
P1	121 ± 8	0.15	−50.3 ± 7	89 ± 6
P2	128 ± 10	0.18	−51.7 ± 10	93 ± 9
P3	132 ± 7	0.17	−48.7 ± 9	91 ± 8

**Table 2:** Transmembrane potential ± SD of control RBCs and RBCs modified by P1, P2 and P3 liposomal formulation in in vitro studies. * Statistically significant differences between control and P1, P2 or P3-treated cells are denoted (α = 0.05).

	Transmembrane Potential of RBCs [mV]
control	−15.6 ± 2.6
P1	−19.6 ± 1.9 *
P2	−20.9 ± 2.1 *
P3	−16.0 ± 1.9

**Table 3:** Average values ± SD of erythrocyte cell stiffness modified with P1, P2, and P3 liposomal formulation. * Statistically significant differences between control and P1, P2, or P3-treated cells are denoted (α = 0.05).

	RBCs Stiffness [pN/μm]
control	32 ± 3
P1	21 ± 4 *
P2	20 ± 4 *
P3	26 ± 3
